# Intracellular A53T Mutant α-Synuclein Impairs Adult Hippocampal Newborn Neuron Integration

**DOI:** 10.3389/fcell.2020.561963

**Published:** 2020-11-11

**Authors:** Martin Regensburger, Judith Stemick, Eliezer Masliah, Zacharias Kohl, Beate Winner

**Affiliations:** ^1^Department of Stem Cell Biology, Friedrich-Alexander University Erlangen-Nürnberg, Erlangen, Germany; ^2^Department of Molecular Neurology, Friedrich-Alexander University Erlangen-Nürnberg, Erlangen, Germany; ^3^Center for Rare Diseases Erlangen (ZSEER), University Hospital Erlangen, Erlangen, Germany; ^4^Department of Neurosciences, University of California, San Diego, La Jolla, CA, United States; ^5^Division of Neuroscience and Laboratory of Neurogenetics, National Institute on Aging, Bethesda, MD, United States; ^6^Department of Neurology, University of Regensburg, Regensburg, Germany

**Keywords:** A53T alpha-synuclein, cell autonomous, adult neurogenesis, hippocampus, spines

## Abstract

Dendritic dysfunction is an early event in α-synuclein (α-syn) mediated neurodegeneration. Altered postsynaptic potential and loss of dendritic spines have been observed in different *in vitro* and *in vivo* models of synucleinopathies. The integration of newborn neurons into the hippocampus offers the possibility to study dendrite and spine formation in an adult environment. Specifically, survival of hippocampal adult newborn neurons is regulated by synaptic input and was reduced in a mouse model transgenic for human A53T mutant α-syn. We thus hypothesized that dendritic integration of newborn neurons is impaired in the adult hippocampus of A53T mice. We analyzed dendritic morphology of adult hippocampal neurons 1 month after retroviral labeling. Dendrite length was unchanged in the dentate gyrus of A53T transgenic mice. However, spine density and mushroom spine density of newborn neurons were severely decreased. In this mouse model, transgenic α-syn was expressed both within newborn neurons and within their environment. To specifically determine the cell autonomous effects, we analyzed cell-intrinsic overexpression of A53T α-syn using a retrovirus. Since A53T α-syn overexpressing newborn neurons exhibited decreased spine density 1 month after labeling, we conclude that cell-intrinsic A53T α-syn impairs postsynaptic integration of adult hippocampal newborn neurons. Our findings further support the role of postsynaptic degeneration as an early feature in synucleinopathies and provide a model system to study underlying mechanisms.

## Introduction

Accumulation of α-synuclein (α-syn) is the neuropathological hallmark of synucleinophathies like Parkinson’s disease (PD), resulting in cytoplasmic inclusions called Lewy bodies (Spillantini et al., 1997; Goedert et al., 2012). While the precise mechanisms of α-syn mediated neurodegeneration are incompletely understood, impaired synaptic transmission was related to axonal degeneration in PD (Chung et al., 2009; Prots et al., 2018).

The A53T α-syn mutation causes early-onset familial PD (Polymeropoulos et al., 1996). Increased α-syn toxicity conferred by this variant was linked to a higher propensity for aggregation when compared to wild-type α-syn and to A30P α-syn (Conway et al., 2000b; Ostrerova-Golts et al., 2000). Specifically, the A53T mutation increases levels of oligomeric α-syn species (Conway et al., 2000a; Winner et al., 2011a; Zambon et al., 2019). In A53T-linked familial PD, non-motor symptoms including cognitive impairment were reported (Puschmann et al., 2009). Post-mortem analyses of A53T PD cases revealed widespread accumulation of insoluble α-syn, including the hippocampus (Duda et al., 2002; Kotzbauer et al., 2004).

Transgenic (tg) animal models overexpressing α-syn have improved the understanding of neurodegeneration in synucleinopathies. Different tg models overexpressing A53T α-syn have been shown to recapitulate parts of the A53T phenotype *in vivo* (Hashimoto et al., 2003; Visanji et al., 2016).

In the mammalian brain, neurogenesis persists throughout adulthood within the so-called neurogenic niches, i.e., the hippocampus and the subventricular zone/olfactory bulb system. This offers the unique opportunity to study the integration of birthdated newly generated neuroblasts into an existing local microcircuitry (Gonçalves et al., 2016). Survival and dendritic integration of newborn neurons are impaired in mice tg for human wildtype α-syn (Winner et al., 2004, 2012; Regensburger et al., 2018). Previously, we have also shown that survival of newborn neurons is significantly reduced in the adult hippocampus of A53T-tg mice which was related to altered cell intrinsic expression of the Notch signaling pathway (Crews et al., 2008; Kohl et al., 2012). In addition, serotonergic innervation was reduced in the A53T-tg mouse model within specific subregions of the prefrontal cortex and the hilus of the hippocampal dentate gyrus, correlating with reduced serotonergic imaging markers in presymptomatic A53T mutations carriers (Deusser et al., 2015; Wihan et al., 2019; Wilson et al., 2019).

The integration of adult newborn neurons provides a useful model to study the effects of disease related proteins on synapse formation in an aging brain (Mu et al., 2010). Our previous data indicate both serotonergic degeneration and impaired neurogenesis in the A53T-tg mouse model. We here aimed to analyze newborn neuron integration within the molecular layer of the adult dentate gyrus. We demonstrate a synaptic integration deficit at the level of spines and mushroom spines. Importantly, we show that cell-intrinsic A53T α-syn is sufficient to impair spine density in non-tg mice. Our findings support emerging evidence about a pathogenic role of α-syn in the postsynaptic compartment.

## Materials and Methods

### Animals

Animal experiments were conducted in accordance with the European Communities Council Directive of 24th Nov. 1996 and were approved by the local governmental administrations for animal health (Animal Care Use Committee of the University of California, San Diego, CA, United States and Government of Lower Frankonia, Würzburg, Germany, “TS–9/11”). A53T-tg mice overexpress human α-syn carrying the A53T mutation under the regulatory control of the PDGFβ promoter (Hashimoto et al., 2003). A53T-tg mice were kept group-housed with non transgenic littermates (non-tg) under a 12 h light dark cycle with free access to food and water. Newborn neurons were labeled with the respective retrovirus at 4 months of age (*n* = 4 animals per group) when survival and proliferation of newborn neurons is impaired (Kohl et al., 2012). For the analysis of the effects of cell-intrinsic overexpression of A53T-mutant α-syn, newborn neurons were labeled in C57Bl/6 mice at the young adult age of 6 weeks (*n* = 4 animals per group).

### Labeling of Newborn Neurons

A Moloney murine leukemia retrovirus-based CAG-GFP plasmid was used as described earlier (Zhao et al., 2006; Winner et al., 2012; Regensburger et al., 2018). To selectively overexpress A53T-mutant α-syn within newborn neurons, the retrovirus CAG-A53T-GFP was cloned where A53T-mutant α-syn is C-terminally fused with GFP. To this end, GFP cDNA was ligated to the 3′ end of A53T-mutant α-syn cDNA in a Bluescript cloning vector (Invitrogen) and subsequently cloned into the CAG-GFP vector. Correct structure of the construct was confirmed by sequencing. A concentrated viral solution (10^8^ pfu/ml) was prepared with human embryonic kidney 293T packaging cells (Tashiro et al., 2006). Mice were anesthetized using a weight-adjusted i.p. dose of Xylazine/Ketamine. A stereotaxic frame (Kopf Instruments) was used for sequential bilateral infusion into the dentate gyrus (AP −2.00 mm, ML +/− 1.6 mm from bregma, DV −2.3 mm from skull). A total volume of 1.5 μl was slowly infused (0.3μl/min) followed by wound closure and a survival period of 31 days.

### Tissue Processing

Animals were sacrificed 31 days after stereotactic surgery. Euthanasia with Xylazine/Ketamine i.p. was followed by transcardial perfusion with PBS and 4% paraformaldehyde for tissue fixation. Brains were dissected, postfixed for 6 h in 4% paraformaldehyde and stored in 30% sucrose in 0.1 M phosphate buffer at 4°C. 40 μm coronal brain sections were stored in cryoprotectant solution (25% ethylene glycol, 25% glycerol in 0.1 M phosphate buffer) at −20°C.

### Immunohistochemistry

The following primary antibodies were used: rat-anti-α-synuclein (15G7, Enzo Life Science, Germany, 1:50), ch-anti-GFP (Abcam, 1:500), rb-anti-SERT (ImmunoStar, Hudson, WI, United States; 1:2,000), gt-anti-DCX (C18, Santa Cruz, TX, United States). Secondary antibodies were donkey-derived and conjugated with Alexa-488 (1:1,000; Life Technologies, Carlsbad, CA, United States), Alexa-647, or Rhodamine Red-X (1:1,000; Dianova). Immunofluorescence was conducted as described previously (Winner et al., 2012; Deusser et al., 2015). Sections were blocked in 3% donkey serum/0.1% TritonX100 in TBS, and incubated with primary antibodies at 4°C, with secondary antibodies at room temperature, and washed again in TBS. Nuclei were counterstained with DAPI (Thermo Fisher Scientific, final concentration 1:2,000) and mounted on object glasses (Superfrost Slides, Menzel).

### Microscopy

Microscopy and dendrite analyses were performed as reported previously (Regensburger et al., 2018). Recordings were performed on a fluorescence microscope (Observer.Z1, Zeiss, for dendrite growth analysis) and on a confocal laser scanning microscope (LSM710, Zeiss for spine analysis and coexpression studies) using ZEN software. For dendrite growth analyses, on average 6 GFP-positive newborn neurons in the dentate gyrus of each animal were imaged resulting in a cell number of 24 per group. For each neuron, z-series of antibody-enhanced GFP-signal at 1.5 μm were acquired spanning the whole extent of the neuron within the section. Maximum intensity projections were then analyzed with the ImageJ plugin “Simple Neurite Tracer.” Primary, secondary, tertiary and quaternary dendrites were labeled semiautomatically. As readouts, the plugin determined total dendritic length, number of branching points and number of each suborder of dendrites. On a thresholded image of the rendered path of each neuron, dendrite complexity was determined using the “Sholl analysis” plugin, with a step radius of 12.5 μm from soma and a maximum radius of 250 μm.

Spine recordings were performed on dendritic sections localized within the molecular layer, using unstained mounted sections and the 60x object lens. Six newborn neurons were analyzed per group. The field of view was placed on the molecular layer and all dendritic segments of one positive neuron localized within this area were imaged. Upon morphological appearance, spines were classified into thin/stubby spines and the small subpopulation of mushroom spines. The estimated surface area of each spine was calculated as 0.785 × *D*_major_ × *D*_minor_ with *D*_major_ as the biggest diameter and *D*_minor_ as the smallest diameter of the respective spine. Mushroom spines exhibit a surface area of at least 0.4 μm^2^ which was measured for each (suspected) mushroom spine (Zhao et al., 2014).

To determine the density of SERT-positive fibers, stacked images of the molecular layer were scanned, spanning a *z*-axis of 11 μm at 1 μm distance. On maximum intensity projection images, SERT-positive fibers were manually traced within the molecular layer using the multipoint line tool in ImageJ. Density was calculated by division of the total SERT length by analyzed area and 11 μm.

### Statistics

All data are shown as mean ± standard deviation except for Sholl analyses (mean ± SEM). Statistical analyses were performed using Graph Pad Prism (GraphPad Software, La Jolla, CA, United States). Statistical significance was indicated by ^∗^*P* < 0.05, ^∗∗^*P* < 0.01, and ^∗∗∗^*P* < 0.001, as determined by unpaired, two-sided *t*-tests.

## Results

### Impaired Spine Morphology of Adult Newborn Neurons in A53T-tg Mice

**FIGURE 1:**
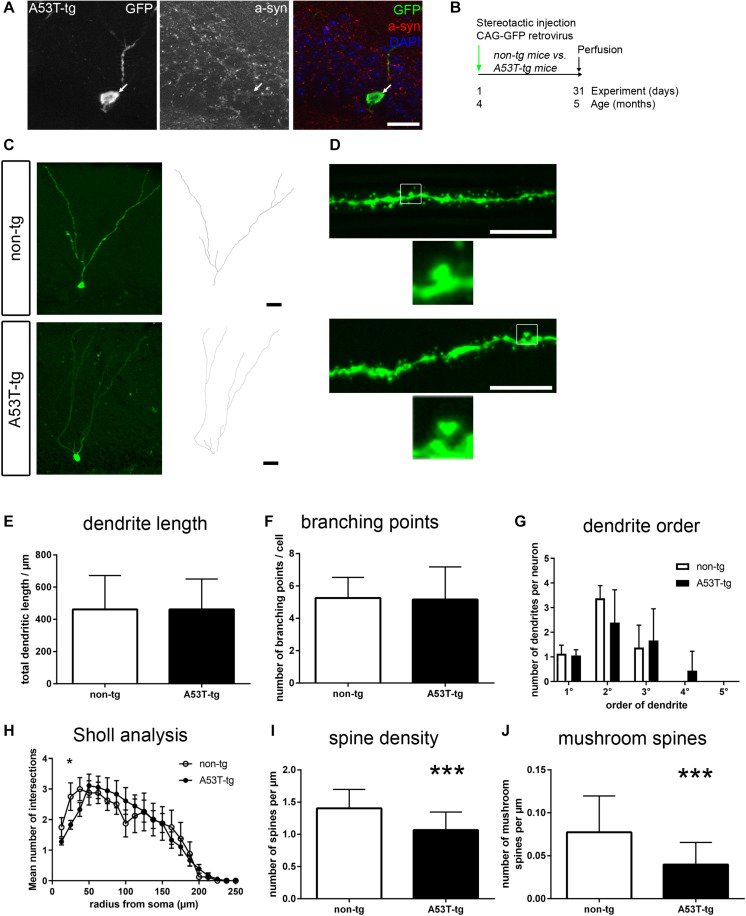
Impaired postsynaptic integration of adult newborn neurons in A53T-transgenic mice. **(A)** In PDGF::A53T-α-syn transgenic animals, A53T-tg α-syn was present in the granule cell layer and colocalized with GFP-positive newborn neurons (arrow). **(B)** Experimental paradigm: in A53T-tg vs. non-transgenic mice, newborn neurons were stereotactically labeled with a GFP expressing retrovirus at the age of 4 months. Perfusion was performed after 31 days. **(C)** Sample tracings of newborn neurons in non-tg and A53T animals. **(D)** Sample micrographs of dendritic spines of newborn neurons. Magnifications show mushroom spines. **(E–G)** Dendrite length, the number of branching points and a quantification of different orders of dendritic branches were unchanged in A53T-tg animals. **(H)** Sholl analysis of dendrite complexity was slightly decreased in A53T-tg at 25 μm from soma, but otherwise unchanged. **(I)** Significant reduction of dendritic spine density in A53T-tg animals. **(J)** Significant reduction of mushroom spine density in A53T-tg animals. For statistical analysis, refer to Table 1. Graphs show mean ± standard deviation except for **(H)** (mean ± SEM). Scale bars 25 μm **(A,C)**, 10 μm (fluorescent images in **D**).

It was previously shown that at the age of 4 months, proliferation and survival of newborn neurons are impaired in A53T-tg mice (Kohl et al., 2012). Thus, we asked whether the morphology of newborn neurons within the A53T-tg α-syn overexpressing microenvironment was changed. To this end, newborn neurons were labeled by stereotactic injection of a GFP overexpressing retrovirus into the dentate gyrus of 4 months old mice (Figures 1A,B). After a survival period of 1 month, newborn neurons were visualized by fluorescence microscopy for GFP (Zhao et al., 2006). Using an antibody specific for human α-syn, we found that transgenic A53T α-syn was widely expressed in the dentate gyrus, including GFP positive newborn neurons (Figure 1A).

We observed no significant differences in the total dendritic length of newborn neurons, comparing A53T-tg and non-tg (Figures 1C,E and Table 1). Similarly, there were also no differences in dendritic complexity, as measured by the number of branching points (Figure 1F) and the subquantification of the numbers of primary, secondary, tertiary and quaternary dendrites (Figure 1G). Sholl analysis showed a significant reduction of dendrite complexity at 25.0 μm distance from soma, but was otherwise unchanged (Figure 1H).

**TABLE 1:** Analysis of neurite morphology of adult newborn neurons in human A53T α-syn transgenic animals (A53T-tg) vs. non-transgenic controls (non-tg), and in C57Bl/6 animals (cell-intrinsic overexpression of A53T α-syn GFP vs. GFP only).

	Non-tg	A53T-tg	*P*	CAG-GFP	CAG-A53T-GFP	*P*
Animals per genotype	4	4		4	4	
Age at injection (months)	4	4		1.5	1.5	
Dendritic length (μm)	462 ± 211	461 ± 189	0.99	631 ± 175	637 ± 214	0.92
Branching points (per cell)	5.25 ± 1.3	5.17 ± 2.0	0.92	6.54 ± 2.19	7.08 ± 1.82	0.35
Spine density (per μm)	1.40 ± 0.29	1.07 ± 0.28	0.0005	1.87 ± 0.59	1.29 ± 0.38	0.004
Density of mushroom spines (per μm)	0.077 ± 0.042	0.040 ± 0.026	0.0008	0.047 ± 0.018	0.035 ± 0.020	0.15
Density of SERT-positive fibers (per μm^3^)	0.019 ± 0.0016	0.018 ± 0.0023	0.43			

*Numbers are given as mean ± SD and respective P-values.*

As an indicator of postsynaptic integration of newborn neurons, we next visualized dendritic spines of GFP labeled newborn neurons in the molecular layer of A53T-tg and non-tg (Figure 1D). Upon quantification, there was a significant, 24% decrease of the density of spines per dendrite length in A53T-tg as compared to non-tg (Figure 1I), indicating an integration deficit of adult newborn neurons in A53T-tg. Since there are only few filopodia and stubby spines on adult newborn granule cells, dendritic spines can be subdivided into thin spines and a smaller proportion of mushroom spines, based upon their morphology (Zhao et al., 2006). Indeed, also the density of mushroom spines was significantly reduced by 48% in A53T-tg (Figure 1J).

In summary, adult newborn neurons were unchanged in A53T-tg regarding dendrite outgrowth, but postsynaptic integration was impaired as marked by decreased densities of overall spine density and mushroom spine density.

### Intact Serotonergic Innervation of the Molecular Layer of A53T-tg Mice

**FIGURE 2:**
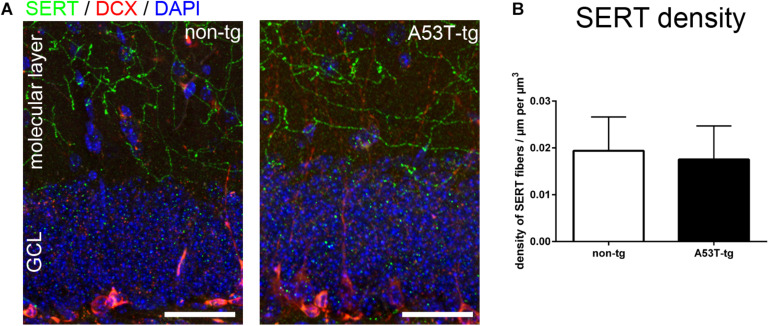
Serotonergic innvervation of the molecular layer of the hippocampus. **(A)** Localization of serotonergic fibers within the molecular layer of the dentate gyrus. **(B)** Quantification of the density of serotonergic fibers within the molecular layer showed no significant differences between non-tg and A53T-tg animals (data included in Table 1). Scale bars 50 μm. *GCL* granule cell layer of the dentate gyrus.

Serotonergic degeneration is a widespread feature in adult A53T-tg mice, including specific layers of the prefrontal cortex and the hilus of the dentate gyrus (Deusser et al., 2015; Wihan et al., 2019). In light of the reduced spine density of newborn neurons, we investigated alterations of presynaptic serotonergic fibers within the molecular layer of adult A53T-tg mice. In accordance with previously published data in a C57Bl/6 based reporter model (Migliarini et al., 2013), there was a dense innervation of the molecular layer both in non-tg mice and in A53T-tg mice (Figure 2A). Upon quantification of the density of serotonergic fibers per volume, no significant differences between A53T-tg and non-tg were present (Figure 2B). This suggests that there is no degeneration of serotonergic fibers within the molecular layer of A53T-tg at this age.

### Specific Effects of Cell-Intrinsic Overexpression of A53T α-Syn on Neuronal Integration

**FIGURE 3:**
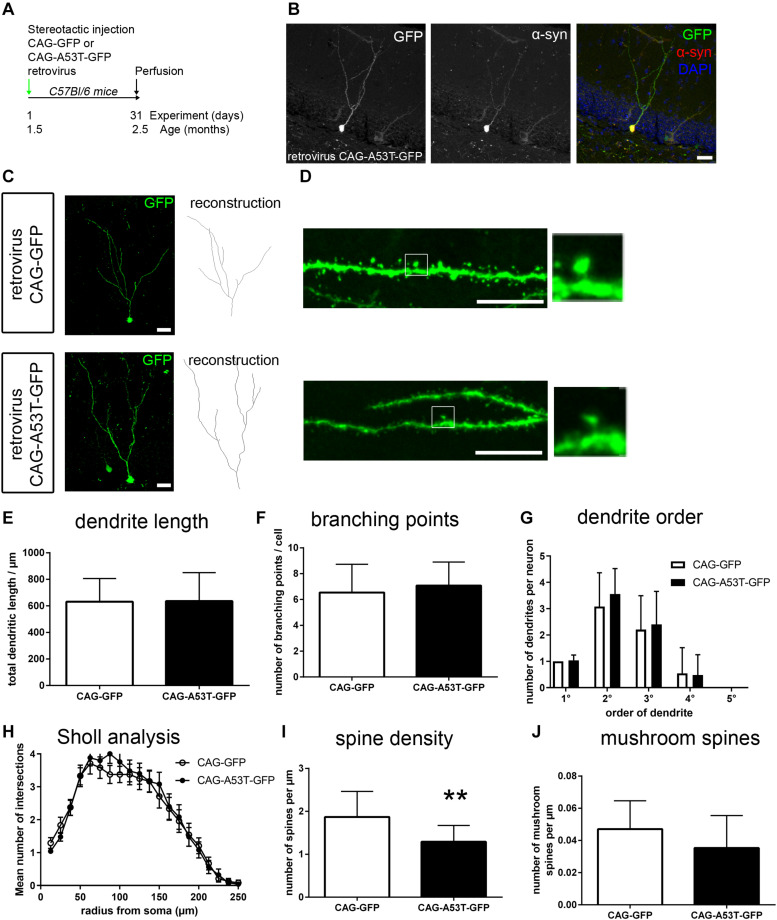
Cell-intrinsic effects of A53T-mutant α-syn on adult newborn neurons. **(A)** Wildtype mice were stereotactically injected into the dentate gyrus, with a CAG retrovirus overexpressing either GFP only or A53T-mutant α-syn and GFP. Perfusion was performed 31 days later. **(B)** Staining with a human specific α-syn antibody showed that overexpressed A53T-mutant α-syn was present within the neuronal soma and the dendritic compartment. **(C)** Sample micrographs of dendritic morphology of GFP labeled newborn neurons. **(D)** Sample micrographs of spines (left) and mushroom spines (magnification on the right) in both groups. **(E–G)** Dendritic length, the number of branching points and numbers of dendrite segments according to order were unchanged in A53T overexpressing neurons. **(H)** Sholl analysis showed no changes upon CAG-A53T-GFP expression. **(I)** Upon cell-intrinsic overexpression of A53T α-syn, the density of spines was significantly reduced. **(J)** Mushroom spine density was not significantly changed. For statistical analysis, refer to Table 1. Graphs show mean ± standard deviation except for **(H)** (mean ± SEM). Scale bars 25 μm **(B,C)**, 10 μm **(D)**.

In order to dissect the role of cell-intrinsic presence of tg A53T α-syn vs. an effect of the transgene within the microenvironment, we next overexpressed A53T α-syn specifically within newly generated neurons. To this end, human A53T α-syn cDNA was cloned into the CAG-GFP retroviral construct. Stereotactic injections of either CAG-GFP or CAG-A53T-GFP were performed in adult wildtype mice, targeting the dentate gyrus of the hippocampus to label newly generated cells (Figure 3A). Perfusion was performed after 31 days. Using a human specific α-syn antibody, we observed that the A53T α-syn GFP fusion protein was localized within the somal, axonal and dendritic compartments of newborn neurons (Figure 3B).

Following the strategy described above for the analysis of A53T-tg mice, we first investigated dendritic morphology (Figure 3C). Cell-autonomous overexpression of A53T-GFP caused no significant differences in dendrite outgrowth, the number of branching points and the number of primary, secondary, and tertiary dendrites when compared to GFP only (Figures 3E–G). Sholl analysis showed that dendrite complexity was unchanged between groups (Figure 3H). This indicates that neurite outgrowth is not impaired by cell-intrinsic presence of A53T α-syn. However, analyzing spine density in CAG-A53T-GFP, we found a significant reduction by 31% as compared to CAG-GFP (Figures 3D,I) which was comparable to the A53T-tg model. Interestingly, mushroom spine density was not significantly reduced upon cell-autonomous A53T-GFP overexpression, although there was a trend (Figure 3J). To sum up, retrovirus based cell-autonomous overexpression of A53T α-syn within the dentate gyrus resulted in decreased overall spine density, but mushroom spine density was not significantly altered.

## Discussion

We report reduced spine density of adult hippocampal newborn neurons (i) in the A53T-tg mouse model and (ii) upon cell-intrinsic overexpression of A53T α-syn. There were no indications of serotonergic axonal degeneration in the molecular layer of A53T-tg mice at this stage. Since we observed early expression of tg A53T α-syn within newborn neurons, our findings suggest the involvement of cell-autonomous effects of A53T α-syn on the impaired postsynaptic integration of newborn neurons into the adult environment.

The observed transgene expression in adult newborn neurons of the A53T-tg model 1 month after labeling matches with previous findings. In A53T-tg mice, α-syn was shown to accumulate within GFAP/Sox2-positive adult stem cells, within DCX positive neuroblasts and within mature NeuN positive neurons (Crews et al., 2008; Winner et al., 2008; Kohl et al., 2012).

Our results add to previous *in vitro* studies showing negative effects of A53T α-syn on neurite growth and synapse formation. Lentiviral overexpression of A53T α-syn in mouse embryonic stem cell derived neurons resulted in reduced neurite growth and reduced levels of β3-tubulin along with increased levels of cell death (Schneider et al., 2007; Crews et al., 2008). In neurons derived from A53T PD patients’ induced pluripotent stem cells, neurite length and the number of synapses was significantly decreased, which might also be caused by postsynaptic presence of α-syn in this model (Kouroupi et al., 2017).

While we have previously described an impaired dendrite growth and dendrite branching upon *in vivo* transduction of newborn neurons with human wildtype α-syn (Winner et al., 2012), our results here show that A53T α-syn specifically impaired spine density. These discrepancies may be caused by different intracellular expression levels of α-syn, e.g., by altered degradation of A53T α-syn when compared to the wildtype form (Stefanis et al., 2001; Cuervo et al., 2004). Alternatively, surviving neurons with unaffected dendritic development may have been selected by early cell death of adult neuroblasts with high levels of A53T α-syn expression (Winner et al., 2008). Due to low infection numbers to enable labeling of single cells, cell survival could not be directly analyzed in our experiments. Since negative effects on dendrite outgrowth and spine formation of adult newborn neurons were also present in a LRRK2^G2019S^-tg mouse model, overlapping mechanisms may be involved (Winner et al., 2011b). Atrophy of dendritic spines due to presynaptic α-syn aggregation is well described in PD brains (Kramer and Schulz-Schaeffer, 2007).

As dendritic spines reflect sites of strongest synaptic input, our data suggest a reduced number and strength of synapses upon cell-autonomous expression of A53T α-syn (Harris and Stevens, 1988). However, a preserved density of synapses despite spine loss cannot be fully excluded based upon our data.

Under physiological conditions, intraneuronal α-syn is mainly localized within axonal presynaptic terminals in fully mature neurons (Withers et al., 1997). During neuronal maturation and under pathological circumstances, however, endogenous α-syn is also located in the somal, nuclear and dendritic compartments (Withers et al., 1997; McLean et al., 2000; Miraglia et al., 2018; Teravskis et al., 2018). Interestingly, the A53T mutation slows axonal transport of α-syn to the presynapse *in vitro* (Yang et al., 2010). Moreover, in A53T-tg mice, dendritic localization of α-syn was ultrastructurally confirmed within the dendrites of mature neurons, predominantly within inclusions (Martin et al., 2006). Thus, the observed dendritic localization of the A53T-GFP fusion protein may also be a result of pathogenic functions of α-syn.

In this study, we specifically analyzed adult newborn neuron integration 1 month after labeling, i.e., shortly after the peak of spine morphogenesis and dendrite growth (Zhao et al., 2006). However, newborn neurons’ spines are constantly generated for at least 2 more months and remain mobile, along with differential synaptic connectivity when compared to fully mature granule cells (Toni and Sultan, 2011; Lemaire et al., 2012; Cole et al., 2020). Thus, we cannot exclude that the observed effects may become more or less severe at later timpoints. However, the 4 weeks timepoint has been commonly used to analyze newborn neuron integration because the selection of surviving newborn neurons is mostly completed (Biebl et al., 2000; Winner et al., 2002; Dayer et al., 2003; Kim et al., 2011; Llorens-Martín et al., 2016). In addition, we have previously shown that altered dendrite and spine morphology upon cell-autonomous overexpression of wildtype α-syn were still present 3 months after labeling (Winner et al., 2012).

A recent long-term follow-up of dendritic trees indicated that hippocampal adult newborn neurons tend to acquire one or more additional primary dendrites after several weeks, probably via displacement of the first branch point of the primary dendrite (Cole et al., 2020). Overexpression of A53T α-syn increased the number of primary neurites in rat midbrain neurons (Koch et al., 2015). In both of the A53T models presented herein, we did not detect changes in the number of primary neurites. In addition, there were no overall changes in dendrite complexity.

The microtubule associated protein tau interacts with α-syn and there is a substantial overlap of tauopathies with synucleinopathies (Moussaud et al., 2014). Of note, A53T α-syn altered the localization of tau within postsynaptic spines of cultured primary neurons (Teravskis et al., 2018). Moreover, memory deficits of A53T-tg mice were absent in a tau-null background (Singh et al., 2019). Tau accumulation was also observed in post-mortem analyses of A53T PD patients (Duda et al., 2002; Kotzbauer et al., 2004). Thus, the observed impairment of spine density and mushroom spine density in A53T-tg mice might be mediated by tau accumulation. Alternatively, increased intracellular levels of A53T α-syn may disrupt spine formation by promoting neuritic mitochondrial dysfunction (Ryan et al., 2013; Kouroupi et al., 2017; Hu et al., 2019). Finally, decreased axonal transport and microtubule stability due to oligomeric α-syn species might have impaired spine genesis or stability (Prots et al., 2013, 2018).

## Conclusion

In summary, we here show that intracellular A53T α-syn disrupts dendritic spine density of newborn neurons in the adult dentate gyurs. As spine morphogenesis and stability are critical for neuronal function, intracellular A53T α-syn may promote dendritic dysfunction by a cell-intrinsic mechanism.

## Data Availability Statement

The raw data supporting the conclusions of this article will be made available by the authors, without undue reservation, to any qualified researcher.

## Ethics Statement

The animal study was reviewed and approved by Animal Care Use Committee of the University of California, San Diego, La Jolla, United States and the Regierung von Unterfranken, Würzburg, Germany.

## Author Contributions

MR, ZK, and BW: conceptualization. MR, JS, EM, and ZK: conduction of experiments, analysis, and interpretation of data. MR: manuscript initial draft. All authors: manuscript critical correction and approval of final version.

## Conflict of Interest

The authors declare that the research was conducted in the absence of any commercial or financial relationships that could be construed as a potential conflict of interest.
